# Supplementary material for: Newborn Screening for the Detection of the TP53 R337H Variant and Surveillance for Early Diagnosis of Pediatric Adrenocortical Tumors: Lessons Learned and Way Forward
Source: Cancers (Basel). 2021 Dec 3;13(23):6111. doi: 10.3390/cancers13236111 (PMC8656743; doi:10.3390/cancers13236111)
Supplement: Supplementary file 1 [file cancers-13-06111-s001.zip › cancers-1381845-supplementary.pdf]

Supplementary materials

Newborn Screening for the Detection of the *TP53* R337H Variant and Surveillance for Early Diagnosis of Pediatric Adrenocortical Tumors: Lessons Learned and Way Forward

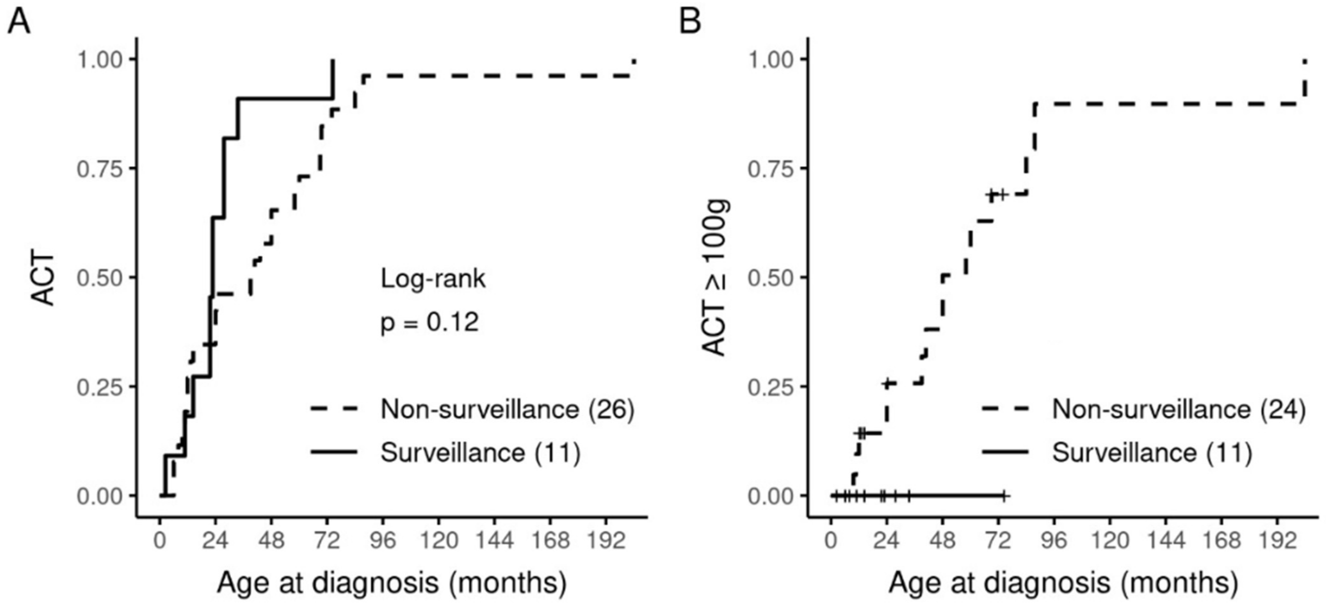

Figure S1. (**A**) There is no significant difference between age at diagnosis of pediatric ACT according to participation in the surveillance. (**B**) Age at diagnosis is strongly associated with tumor weight. The number of children with tumor weight < 100 g is significantly higher in the surveillance group than that of children who did not participate in the surveillance.

Table S1. Features at diagnosis of children with ACT in newborn screening, but not surveillance.

Neonatal Screening	Age (Years)	Stage	Interval Between Symptoms and Diagnosis (weeks)	Tumor Weight (g)	Treatment
1	0.5	I	NA	40	Surgery
2	0.9	II	NA	160	Surgery
3	0.8	II	NA	580	Surgery + Chemotherapy
4	0.5	I	NA	65	Surgery
5	1.2	III (spillage)	NA	90	Surgery + Chemotherapy
6	1.1	I	NA	60	Surgery
7	3.6	III	NA	NA	Surgery + Chemotherapy
8	3.4	III	NA	780	Surgery + Chemotherapy
9	7.3	II	NA	267	Surgery
10	5.8	IV	NA	NA	Unknown
Median	1.2	NA	NA	125	-

Table S2. Analysis of tumor weight according to newborn screening and surveillance.

Tumor Weight (g)	No Newborn Screening or Surveillance (N = 16)	Newborn Screening and No Surveillance (N = 10)	Newborn Screening and Surveillance (N = 11)	p-Value [¥]
<55	5 (31.5%)	4 (40%)	11 (100%)	0.0005 [†]
≥100	11 (68.5%)	6 (60%) *	0	
Median and range (g)	210 (18–608)	125 (40–780)	21 (1–54)	0.0002 [‡]

* Tumor weight was not available but estimated to be greater than 100 g by imaging studies. [¥] The newborn screening and surveillance groups were used as a reference. Data from the other two groups were combined for the analysis. [†] Fisher's exact test. [‡] Kruskal–Wallis test.

Table S3. Analysis of disease stage according to newborn screening and surveillance.

Disease Stage	No Newborn Screening or Surveillance (N = 16)	Newborn Screening and No Surveillance (N = 10)	Newborn Screening and Surveillance (N = 11)	p-Value [¥]
I	5 (31.5%)	3 (30%)	11 (100%)	0.0008 [†]
II–III	6 (37.0%)	6 (60%)	0	
IV	5 (31.5%)	1 (10%)	0	

[¥] The newborn screening and surveillance groups were used as a reference. Data from the other two groups were combined for the analysis. [†] Fisher's exact test.

Table S4. Analysis of duration of symptoms according to newborn screening and surveillance.

Duration of Symptoms	No newborn Screening or Surveillance (N = 16)	Newborn Screening and Surveillance (N = 11)	p-Value [¥]
Median and range (weeks)	17 (3–52)	3 (3–3)	0.00001962 [‡]

[¥] The newborn screening and surveillance groups were used as a reference. There is no available data on the duration of symptoms in children who refused surveillance. [‡] Kruskal-Wallis test.

Table S5. Cost with chemotherapy or surgery for patients with advanced-stage disease.

Patient	Disease Stage	Sites	Expenditures per Year from Diagnosis, in US Dollars										Status
			1st	2nd	3rd	4th	5th	6th	7th	8th	9th	10th	
1	IV	liver	11,289	11,927	6035	169							Dead
2	IV	Lung, liver, bone	6134										Dead
3	IV	Lung	14,852	5967	332	85	12						A, NED
4	II/IV	Lung	3208	4708	9900	5887	168						Dead
5	IV	Lung	10,758	7534	1297	3409	2689	587	587	28	82	8.47	A, NED
6	III		11,928										Dead
7	II/III		645	314	2768	4310	2898	326	173	83	83		A, NED
8	III		7584	2009	193	5404	4890	5282	8021	4454	8955	322	A, NED

A, alive; NED, no evidence of disease.
